# Analysis of circRNAs profile in TNF-α treated DPSC

**DOI:** 10.1186/s12903-022-02267-2

**Published:** 2022-07-03

**Authors:** Qiyin Lei, Zezi Liang, Qiaoling Lei, Fuying Liang, Jing Ma, Zhongdong Wang, Shoudi He

**Affiliations:** 1 Stomatology and Cosmetic Dentistry Center, Shenzhen Hospital of Southern Medical University, Shenzhen, 518000 Guangdong China; 2 Traditional Chinese Medicine Department of Rheumatism, Huazhong University of Science and Technology Union Shenzhen Hospital, No.89 Taoyuan Road, Nanshan District, Shenzhen, 518052 Guangdong China

**Keywords:** hsa_circ_0001978, hsa_circ_0004417, DPSCs, circRNAs profile, Inflammation progress of pulpitis

## Abstract

**Background:**

Pulpitis often are characterized as sustained inflammation and impaired pulp self-repair. Circular RNAs (circRNAs) have been reported to be involved in the development of inflammation, but their influence in pulpitis is still unidentified, which was examined in our research.

**Methods:**

In this study, TNF-α (20 ng/mL) was used to treat DPSCs, then MTS identified cell proliferation. The circRNAs profile in DPSCs with or without TNF-α treatment was evaluated using RNA sequencing and subsequently by bioinformatics analysis. After that, the circular structure was assessed using agarose gel electrophoresis, followed by Sanger sequencing. And the circRNAs expression was ratified using quantitative real-time polymerase chain reaction in cell and tissues samples. Additionally, the plausible mechanism of circRNAs was envisaged, and the circRNA-miRNA-mRNA linkage was plotted using Cytoscape.

**Results:**

The treatment of TNF-α inhibited cell proliferation capabilities in DPSCs, which also made 1195 circRNA expressions undergo significant alterations. Among these changes, 11 circRNAs associated with inflammation were chosen for circular structure verification, and only seven circRNAs (hsa_circ_0001658, hsa_circ_0001978, hsa_circ_0003910, hsa_circ_0004314, hsa_circ_0004417, hsa_circ_0035915, and hsa_circ_0002545) had circular structure. Additionally, five circRNAs expressions (hsa_circ_0001978, hsa_circ_0003910, hsa_circ_0004314, hsa_circ_0004417, and hsa_circ_0035915) had significantly altered between with or without TNF-α treated DPSCs. Furthermore, hsa_circ_0001978 and hsa_circ_0004417 were increased in patients suffering from pulpitis. Furthermore, their ceRNA linkage and Kyoto Encyclopedia of Genes and Genomes analysis suggested that these two circRNAs may participate in the inflammation development of pulpitis via mitogen-activated protein kinase and the Wnt signaling pathway.

**Conclusion:**

This study revealed that the circRNAs profile was altered in TNF-α treated DPSCs. Also, hsa_circ_0001978 and hsa_circ_0004417 may be involved in the inflammation progress of pulpitis. These outcomes provided the latest information for additional research on pulpitis.

**Supplementary Information:**

The online version contains supplementary material available at 10.1186/s12903-022-02267-2.

## Introduction

Pulpitis, also known as dental pulp inflammation, is usually caused by microbe [1, 2]. Thus, its features include persistent inflammation and impaired pulp self-repair [3]. Furthermore, pulpitis is accompany with pain, and as a result, patients with the condition constitute the highest percentage of dental emergencies in both private dental clinics and hospitals [4]. Currently, drilling or filling are utilized for reversible pulpitis treatment, whereas root canal and crown or extraction are applied for inrreversible pulpitis [5]. Nevertheless, these treatments may lead to post-operative discomforts, injuries on the periodontium due to micro-leakages from the tooth crown, and vertical root breakage as treated teeth are more fragile than non-treated teeth [6, 7]. Hence, it is expedient to discover a new technique to mitigate the scourge of pulpitis.

The dental pulp comprises nerves, blood vessels, odontoblasts, fibroblasts, and dental pulp stem cells (DPSCs). DPSCs are profuse cell groups in the dental pulp that promote host resistance and tissue redevelopment [3, 8]. Additionally, DPSCs have been shown to have the same effects as mesenchymal stem cells, such as multi-lineage differentiation, self-replacement ability, and clonogenic assay [9]. Therefore, DPSCs are a powerful tool for the treatment of osteoarthritis [10], Sjögren’s syndrome [11], and pulp self-replacement therapy [12, 13]. Furthermore, pulp regeneration can improve the viability of the dental pulp and even the whole tooth by differentiating into odontoblast-like cells and synthesizing reparative dentin [14, 15]. Therefore, DPSCs perform a crucial function in pulpitis.

Circular RNAs (circRNAs) are a type of RNA with a circular shape formed by back-splicing [16]. Numerous circRNAs have been observed to perform an essential role in varying biological development via functioning as a microRNAs (miRNAs) sponge to control mRNAs expressions, such as differentiation, cell proliferation, immunity feedback, and angiogenesis [17, 18]. Recently, circRNA124534, circ_0026827, circRNA SIPA1L1, and exosomal circLPAR1 were reported to influence the osteogenic differentiation of DPSCs by controlling miRNAs expression [19–22]. However, the role of circRNAs in DPSCs from pulpitis has not yet been described.

Previous studies have discovered that 20 ng/mL TNF-α treated DPSCs influenced inflammation responses [7, 23]. As a result, 20 ng/mL TNF-α was utilized to treat DPSCs in this study, then the circRNAs profile was ascertained using RNA sequencing followed by bioinformatics analysis. Therefore, this study suggests the latest information to further explore the future mechanism of pulpitis and a current understanding for tackling pulpitis.

## Methods and materials

### Cells cultivation and treatment

DPSCs were obtained from Cellcook (Guangzhou, China) with surface marker negative for CD34, CD45, and HLA-DR, while positive for CD90, CD105, and CD146. Also, cells at 3–5 passages were utilized in this research. DPSCs were cultured in DMEM (Gibco, Grand Island, NY, USA), consisting of 10% fetal bovine serum (FBS, Gibco) and 1% antibiotic–antimycotic solution (Invitrogen, Carlsbad, CA, USA) in a humidity vessel with 5% CO_2_ and 95% air at 37 °C. The protocol used for TNF-α treatment was hinged on earlier studies with minimal modification [23]. First, 2 × 10^5^ DPSCs were introduced into six well plates. After that, the medium was separated and inoculated with fresh MEM without FBS when the confluence reached 80% for 24 h. After that, the medium was substituted by MEM augmented with 20% FBS and TNF-α (20 ng/mL) and stored for 48 h. Finally, the cells were harvested for additional examination. The negative control (NC) DPSCs were examined with TNF-α treatment protocol, although ddH_2_O was used instead of TNF-α solution.

### 3-(4,5-Dimethylthiazol-2-yl)-5-(3-carboxymethoxyphenyl)-2-(4-sulfophenyl)-2H-tetrazolium (MTS) assay

After treatment, DPSCs were harvested and placed into 96-well plates in a total number of 5000 cells per well. After that, cell proliferation was identified at 24, 48, and 72 h according to the method of MTS reagent (Biovision, Milpitas, CA, USA). The OD value was recorded at 490 nm.

### RNA sequencing

After treatment, DPSCs were harvested for complete RNA extraction using TRIzol (Invitrogen). After that, the total RNA was used for cDNA libraries formation and sequencing in Novogen (Beijing, China). After that, the RNA sequencing was analyzed using the R package. The probability levels *P* < 0.01 and |log2Ratio|≥ 1 were significantly different between the two groups. The prototype genes of differential expression of circRNAs or mRNAs were used for the Kyoto Encyclopedia of Genes and Genomes (KEGG) pathway analysis using DAVID (https://david.ncifcrf.gov/).

### Construction of circRNA-miRNA-mRNA network

The binding site of circRNAs to miRNAs, and miRNAs to mRNAs were predicted on miRanda. After that, the circRNA-miRNA-mRNA network was plotted using Cytoscape (https://cytoscape.org/).

### Quantitative real-time PCR (qRT-PCR)

The total RNA was detached from the DPSCs or dental pulp using TRIzol (Invitrogen) in this method. After that, 2 µg of total RNA were rearranged into cDNA using PrimeScript™ RT reagent Kit (Takara, Tokyo, Japan) following the manufacturer’s guidelines. Also, qPCR was performed on ABI 7500 (ABI, Foster City, CA, USA) following the method of TB Green Fast qPCR Mix (Takara). The primer information is displayed in Table 1. GAPDH was used as an internal reference.

**Table 1:** Primers sequence used in this study

Primer name	Sequence (5′-3′)
hsa_circ_0001658-CF	CATGCGTCCCCTCATCTCTC
hsa_circ_0001658-CR	GTCCCCTCTGTTGAACCTTCA
hsa_circ_0001978-CF	GACTACAGGTGCTTGCCACT
hsa_circ_0001978-CR	ACTGCTGCAGTGGTCAACTT
hsa_circ_0003910-CF	AGGCTATAAGCTTCTTGAAGGCA
hsa_circ_0003910-CR	AGGACTCTGGAACGTCTGGA
hsa_circ_0004314-CF	AAGACAGCCGATTCACCAGC
hsa_circ_0004314-CR	CAGTAAGCACTTGACACATGACA
hsa_circ_0004417-CF	AGAAGCATCTTGGATCTTACTATTTGG
hsa_circ_0004417-CR	TGTTCTGGGCAGTCATTGGT
hsa_circ_0035915-CF	GCTAAGGAAGAAGAGCGCCT
hsa_circ_0035915-CR	GGTCTAAGAACTCCAGGTGAAA
hsa_circ_0002545-CF	GCCCTTGTGGATAAGCACAAAG
hsa_circ_0002545-CR	GAGGTAAGAGGGGGCTGTCG
hsa_circ_0001978-LF	GCCTCCCAAAGTGCTGAGAT
hsa_circ_0001978-LR	ACTTGTGGGGAGCACTTAGG
hsa_circ_0003910-LF	TCACCGTGTTAGCCAGGATG
hsa_circ_0003910-LR	GTTCTCACCAGAGGCTCACC
hsa_circ_0004314-LF	AGACTTTCCCACAGCTTGCA
hsa_circ_0004314-LR	TGGAACCAGATCATGACTCTCC
hsa_circ_0001658-LF	TGGTGTCACCCTGAGATAGAGA
hsa_circ_0001658-LR	CCGAAGTAACTGATGGCACCT
hsa_circ_0035915-LF	CAAGGACATGGTGCCAAAGG
hsa_circ_0035915-LR	GCCAAAAACAGTGGTCGCTT
hsa_circ_0004417-LF	CTTGGGCAGAAGAGACAAACTC
hsa_circ_0004417-LR	CCATTTCTCCTGGCAGCTTTG
hsa_circ_0002545-LF	CTTTGGGTGTGGGAATGCAG
hsa_circ_0002545-LR	TGGGCCAAGTTTTGAAAGGG
GAPDH-CF	
GAPDH-CR	
GAPDH-LF	
GAPDH-LR	

### Circular structure validation

Divergent primer and convergent primer were produced by Sangon (Shanghai, China), and the detailed information is shown in Table 1. First, the gDNA was extracted from DPSCs using PureLink™ Genomic DNA Mini Kit (Invitrogen). After that, PCR was carried out by using the method of Platinum™ II Hot-Start Green PCR Master Mix (Invitrogen). Then, the products were further electrophoresed using 2% agarose gel, in which the effects from divergent primer were additionally subjected to Sanger sequencing in Sangon.

### Collection of clinical sample

The study was endorsed by the ethics committee of Shenzhen Hospital, Southern Medical University (endorsement code: NYSZYYEC20190024). The consent letters were received from seven patients suffering from pulpitis and nine others suffering from orthodontic. The diagnostic requirement for pulpitis includes the following: (1) spontaneous pain, nocturnal pain; (2) carious pulp exposure; (3) post-pulpotomy wherein the pulp becomes dark red and bleeds out. As a result, the bleeding is difficult to stop. Exclusion requirements for pulpitis include the following: (1) fistula and abscess can be observed at the root tip; (2) X-ray examination showing a large area of transmission shadow at the root tip or root bifurcation. The diagnostic measures for orthodontic include the following: (1) young permanent teeth with open paramount foramen; (2) supernumerary tooth. Furthermore, the exclusion criteria for orthodontic include the following: (1) young permanent teeth with caries or pulp inflammation or periapical injuries; (2) accidental exposure of young permanent teeth due to trauma; (3) the presence of a deformed central tip. Dental pulp from pulpitis was collected using the protocol as described. First, the infected pulp was extracted with the help of a pulp extraction needle after pulp opening. After that, the samples were stored at − 80 °C for additional analysis. Furthermore, dental pulp from orthodontic (control group) was harvested as described. After tooth extraction, it was washed with normal saline to remove excess blood and soft dirt. Then, the pulp was removed entirely with a pulp extraction needle and stored at − 80 °C for additional examination.

### Statistical analysis

Data were examined using the Graphpad 7.0 statistical tool (La Jolla, CA, USA), and the results were represented as mean ± standard deviation. T-test was used to distinguish the variance between the two groups, and a *P* value less than 0.05 was regarded as statistically significant.

## Results

### CircRNAs expression was changed in TNF-α treated DPSCs

**Fig. 1:**
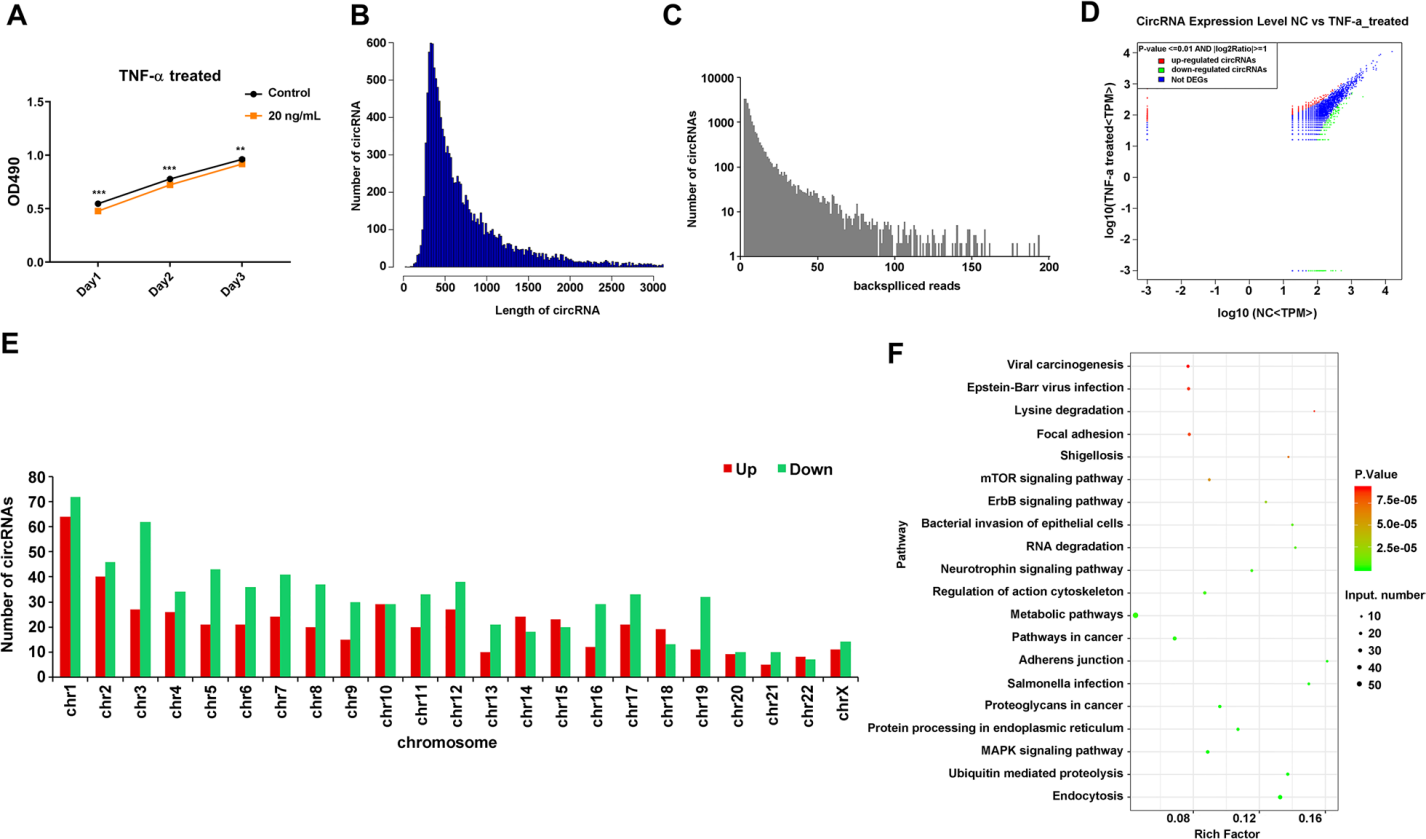
CircRNAs expression were changed in TNF-α treated DPSCs. **A** The proliferation of DPSCs in TNF-α (20 ng/ml) treated DPSCs and negative control (NC) DPSCs was identified using MTS assay. N = 3, T-test; **, indicates *P* value less than 0.01; ***, indicates *P* value less than 0.001. **B** The length distribution of circRNAs was shown in the column chart. **C** The circRNAs’ backspliced was shown in the column chart. **D** Differentially expressed circRNAs between TNF-α treated group, and the NC group were shown in the heat map. **E** The up- and down-regulated circRNAs’ distribution on the chromosome was shown in the column chart. **F** The top 20 KEGG pathways of differentially expressed circRNAs’ parent genes were shown in a bubble chart

To examine the fluctuation of the circRNAs profile in DPSCs after treating inflammatory factors, TNF-α (20 ng/ml) was used to treat DPSCs for 48 h [7, 23]. After that, the cell proliferation was determined at 24, 48, and 72 h. As shown in Fig. 1A, the cell proliferation significantly decreased in TNF-α treated group compared with the NC group. After that, circRNAs profile was obtained using RNA sequencing. The size of circRNAs mainly ranged from 100 to 2500 bp, and the majority were about 500 bp (Fig. 1B). Additionally, most circRNAs’ backspliced reads ranged 1–100 (Fig. 1C). Furthermore, there were 1195 differential expression circRNAs (*P* < 0.01, |log2Ratio|≥ 1) in TNF-α treated group compared with those in the NC group, in which 487 circRNAs were up-regulated and 708 circRNAs were down-regulated (Fig. 1D). The top 3 of the up-regulated circRNAs’ distribution on the chromosome were chr 1, chr 2, and chr 10, and the top 3 of the down-regulated circRNAs’ distribution on the chromosome were chr 1, chr 2, and chr 3 (Fig. 1E). The original gene of the differentially expressed circRNAs was further for the KEGG analysis. The top 20 pathways were represented in Fig. 1F, in which the mitogen-activated protein kinase (MAPK) pathway has been reported to participate in inflammation development [24, 25]. The above outcomes have indicated that many circRNAs expressions have been altered in DPSCs by TNF-α treatment.

### The expression of circRNAs and its circular structure validation

**Fig. 2:**
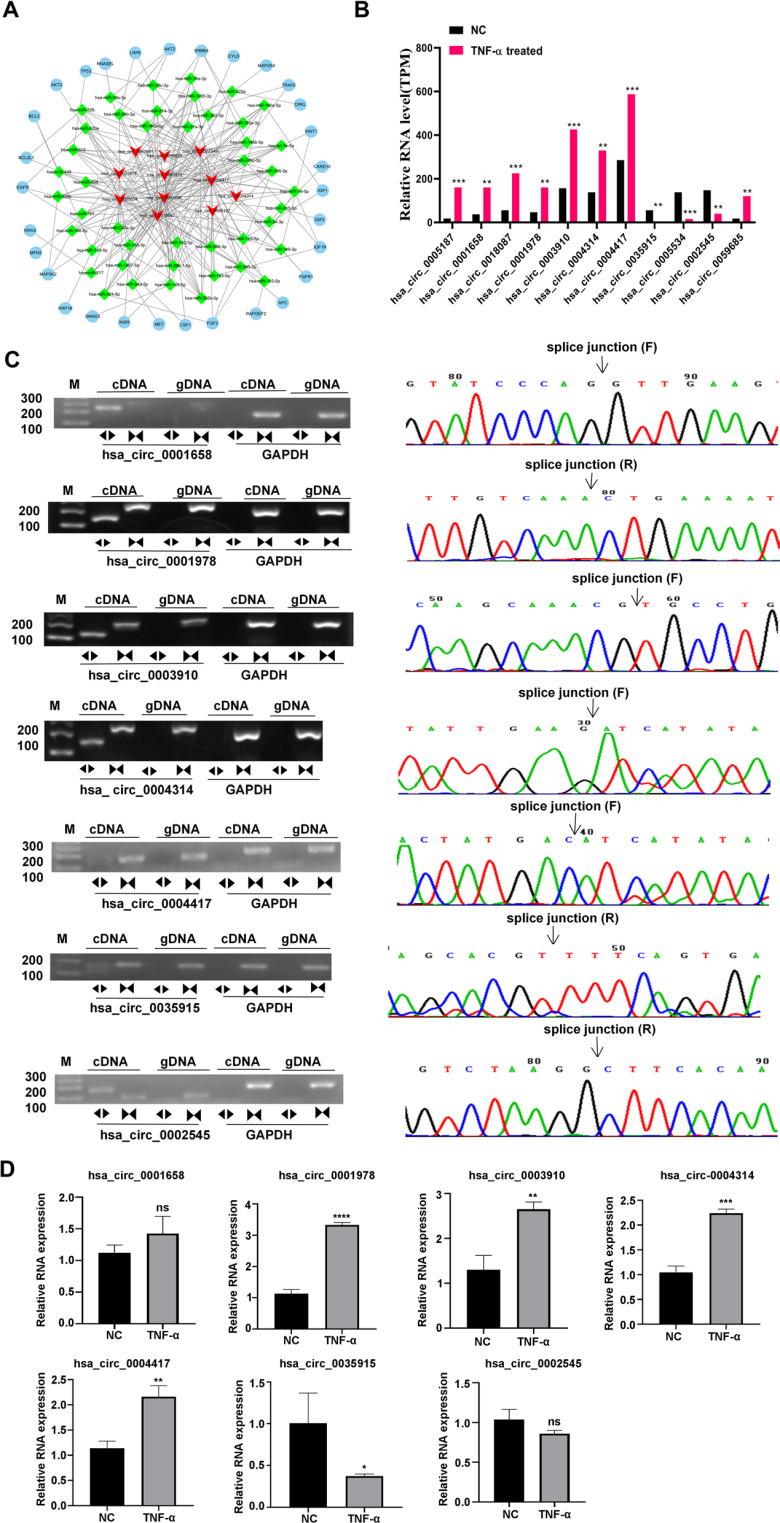
The expression of circRNAs in DPSCs and their circular structure validation. **A** The circRNA-miRNA-mRNA linkage was plotted using Cytoscape; red represented circRNA, green represented miRNA, and blue indicated mRNA. **B** The expression changes of these 11 circRNAs in RNA sequencing data were shown in the column chart. N = 3, T-test; **, indicates *P* value less than 0.01; ***, indicates *P* value less than 0.001. **C** The circular structure was verified using agarose gel electrophoresis and Sanger sequencing. The original blots were included in a Additional file 1, in which the edges with no signals were not included. **D** The expression of circRNAs was identified using the qRT-PCR protocol in TNF-α treated DPSCs and NC DPSCs. N = 3, T-test; ns, indicates no difference; *, indicates *P* value less than 0.05; **, indicates *P* value less than 0.01; ***, indicates *P* value less than 0.001; ****, indicates *P* value less than 0.0001

To further verify which circRNAs participated in the occurrence of pulpitis, the circRNA-miRNA-mRNA linkage was plotted using Cytoscape, in which the differential expression circRNAs were chosen by adhering to these guidelines: (1) the number of envisaged binding locations of circRNAs to miRNAs, and miRNAs to mRNAs was more than three; (2) The gene was envisaged to participate in pulpitis linked signaling pathway, including TNF, NF-κB, NLRP3 inflammasome, MAPK, and Wnt signaling pathways [25–28]. As shown in Fig. 2A, there were 11 circRNAs, 44 miRNAs, 30 mRNAs were discovered and plausibly involved in the development of pulpitis. The expression alterations of these 11 circRNAs in RNA sequencing data were presented in Fig. 2B, the expression of hsa_circ_0005187, hsa_circ_0001658, hsa_circ_0018087, hsa_circ_0001978, hsa_circ_0003910, hsa_circ_0004314, hsa_circ_0004417, and hsa_circ_0059685 were all augmented in TNF-α treated DPSCs than that in the NC group; the expression of hsa_circ_0035915, hsa_circ_0005534, and hsa_circ_0002545 all declined in TNF-α treated DPSCs compared with NC group. Then the circular structure of these 11 circRNAs was verified using agarose gel electrophoresis and Sanger sequencing. The outcomes indicated that seven circRNAs (hsa_circ_0001658, hsa_circ_0001978, hsa_circ_0003910, hsa_circ_0004314, hsa_circ_0004417, hsa_circ_0035915, hsa_circ_0002545) had circular structure; their divergent primers products only were amplified in cDNA, which was used to verify the junction site by Sanger sequencing (Fig. 2C). These seven circRNAs expressions were subsequently validated in RNA sequencing samples. The outcome proves that hsa_circ_0001978, hsa_circ_0003910, hsa_circ_0004314, and hsa_circ_0004417 were significantly improved, hsa_circ_0035915 was notably decreased in TNF-α treated DPSCs than NC group (Fig. 2D). The above results were in harmony with RNA sequencing outcomes. Generally, 5 circRNAs expressions (hsa_circ_0001978, hsa_circ_0003910, hsa_circ_0004314, hsa_circ_0004417, and hsa_circ_0035915) had notably changed between TNF-α treated DPSCs and NC group, which may likely be implicated in TNF, NF-κB, NLRP3 inflammasome, MAPK, and Wnt signaling pathways.

### hsa_circ_0001978 and hsa_circ_0004417 were up-regulated in pulpitis patients

**Fig. 3:**
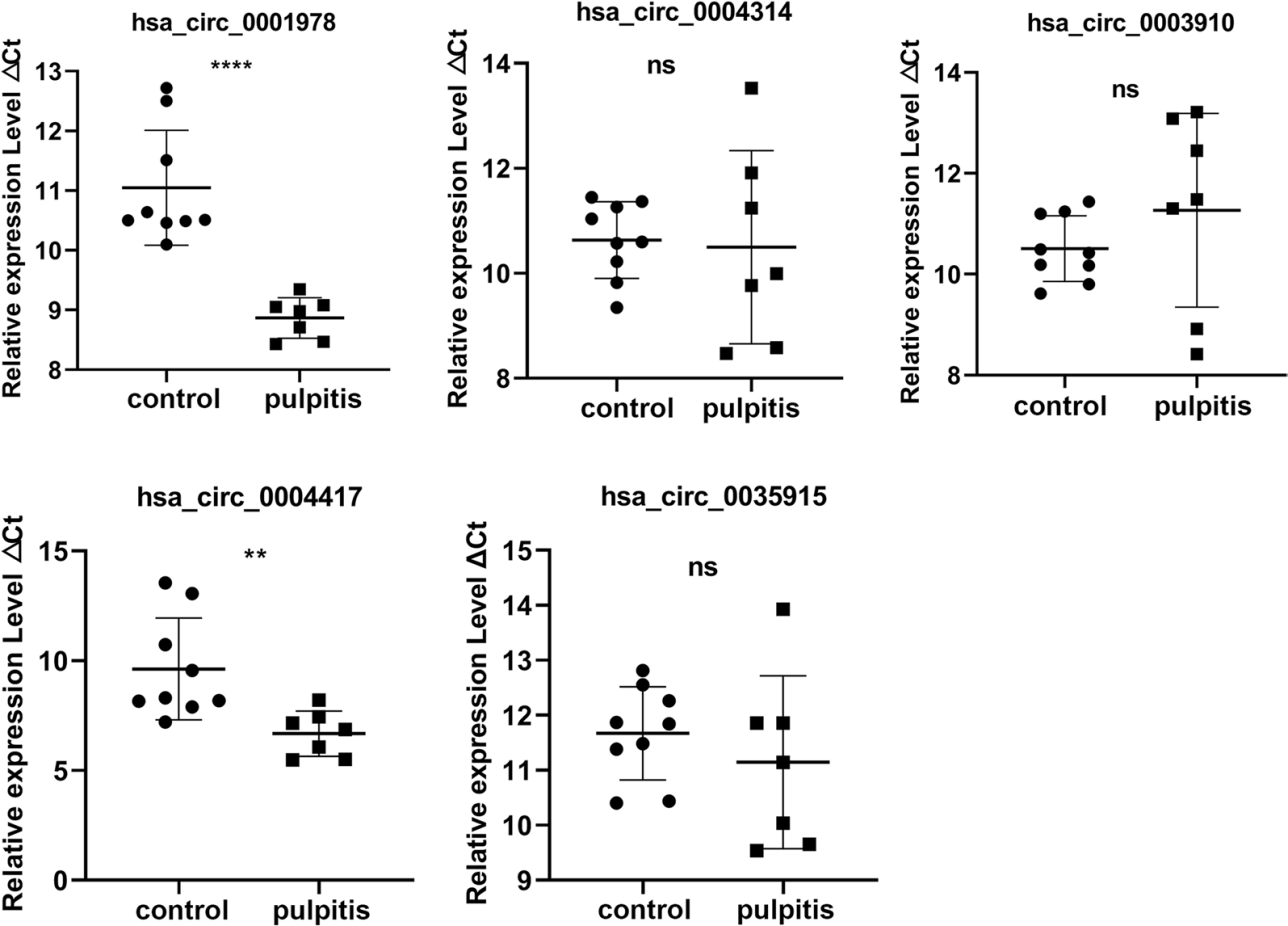
The expression of circRNAs in dental pulp tissue. The expression of hsa_circ_0001978, hsa_circ_0003910, hsa_circ_0004314, hsa_circ_0004417, and hsa_circ_0035915 were identified in dental pulp from patients with pulpitis or orthodontic using qRT-PCR methods. N = 3, T-test; ns, indicates no difference; **, indicates *P* value less than 0.01; ****, indicates *P* value less than 0.0001

The five circRNAs expressions were subsequently confirmed in dental pulp tissue. As shown in Fig. 3, hsa_circ_0001978 and hsa_circ_0004417 expression had variance between pulpitis samples and control samples obtained from patients with orthodontic. As delta Ct value increased, the expression declined, and as a result, their expression behavior was in harmony with RNA sequencing. These outcomes suggested that hsa_circ_0001978 and hsa_circ_0004417 may plausibly perform a crucial role in pulpitis.

### hsa_circ_0001978 and hsa_circ_0004417 maybe regulate MAPK and Wnt signaling pathways after TNF-α treatment in DPSCs

**Fig. 4:**
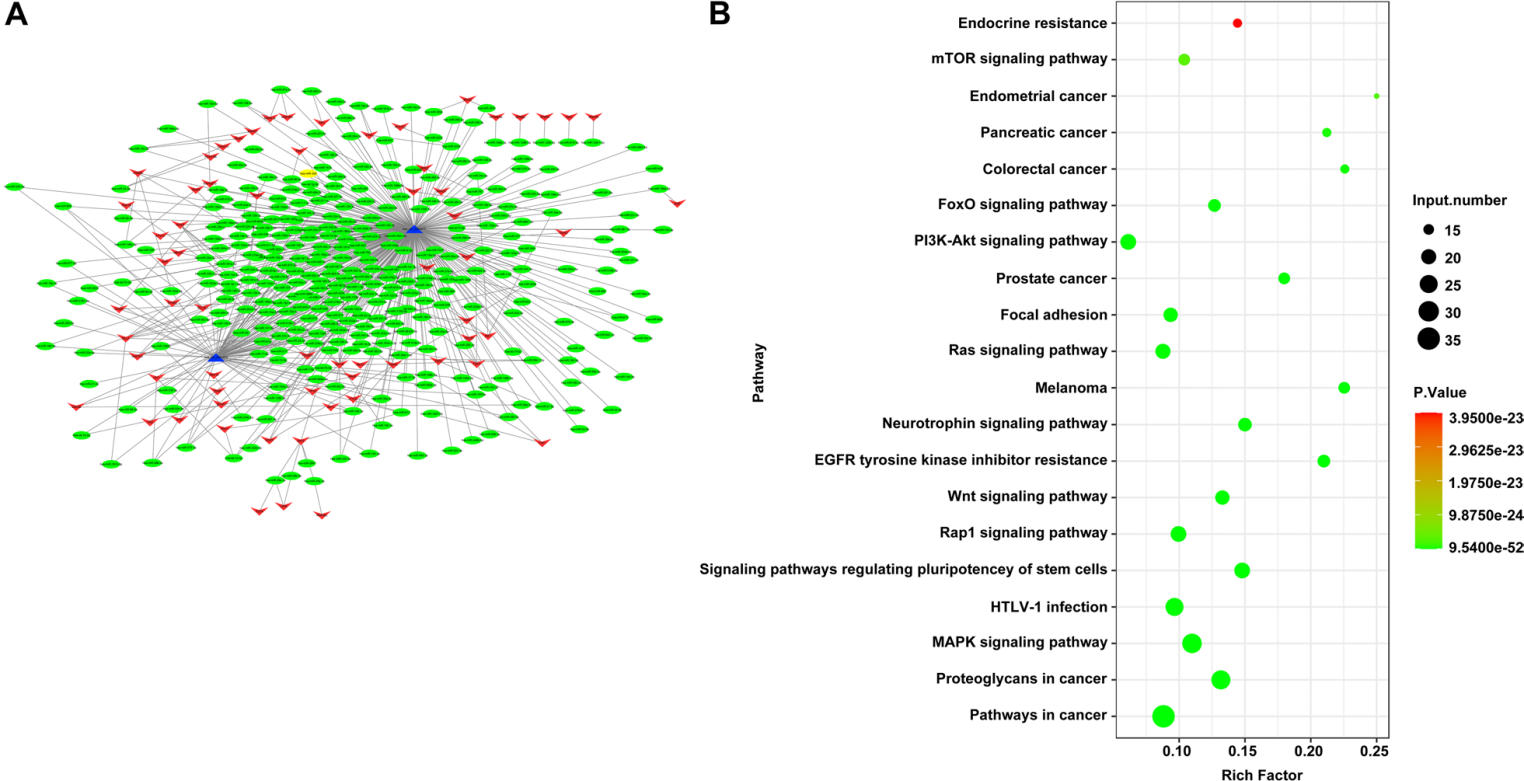
Hsa_circ_0001978 and hsa_circ_0004417 maybe regulate MAPK and Wnt signaling pathways in TNF-α treated-DPSCs. **A** The circRNA (hsa_circ_0001978 and hsa_circ_0004417)-miRNA-mRNA linkage was plotted using Cytoscape; blue represented circRNA, green represented miRNA, and red indicated mRNA. **B** mRNAs in the network of (**A**) were used to carry out KEGG analysis

To further elucidate the prospective mechanism of hsa_circ_0001978 and hsa_circ_0004417, a new circRNA (hsa_circ_0001978 and hsa_circ_0004417)-miRNA-mRNA network was constructed based on a prediction by using miRanda. As presented in Fig. 4A, 396 miRNAs and 65 mRNAs were implicated. Then mRNAs in this linkage were used in KEGG analysis, MAPK and Wnt signaling pathways remained in the top 20 pathways (Fig. 4B). The above results proved that hsa_circ_0001978 and hsa_circ_0004417 may influence TNF-α-treated-DPSCs via MAPK and Wnt signaling pathway.

## Discussion

Pulpitis is a common infection of dental pulp tissue [29]. Also, immune and non-immune cells, cytokines, and chemokines were regarded as modulating factors in pulpitis [30]. Therefore, the principal treatment measures of pulpitis are based on the exclusion of inflamed or necrotic pulp tissue and substitution with a synthetic biomaterial [31]. Recently, complete or incomplete pulp regeneration has been suggested as an alternate therapeutic concept. DPSCs were regarded as an efficient constituent due to their function in tooth development, healing, rejuvenation, and immunomodulatory processes [15, 32]. However, the progression of pulpitis is responsible for developing DPSCs with diminished immunosuppressive capacity. This action is induced by calcitonin gene-related peptide to release proinflammatory cytokines and chemokines, which stimulate neuronal sensitization and contribute to the discomfort of pulpitis [32, 33]. Furthermore, DPSCs may lose their potential for odontogenic regeneration due to the progression of pulpitis [34]. Therefore, redefining the role of DPSCs in pulpitis may be a method to tackle pulpitis.

CircRNAs were regarded as biomarkers and therapeutic targets of human cancer [35]. However, there are no reports regarding their application as therapeutic targets in pulpitis. In this study, it was discovered that DPSCs cells proliferation efficacy declined after TNF-α treatment. Additionally, it was discovered that 1195 circRNA expressions were altered in TNF-α treated DPSCs. Among these circRNAs, hsa_circ_0001978 and hsa_circ_0004417 may likely control the inflammation progression of pulpitis via MAPK and Wnt signaling pathway.

RNA sequencing is a protocol that affords additional information for subsequent studies. CircRNA sequencing is a form of RNA sequencing, which provides new circRNAs for subsequent molecular mechanism examination. For instance, a new circRNA circDENND1B characterized by circRNA sequencing was discovered to control the interface between inflammation and cholesterol transport through miR-17-5p/Abca1 axis in atherosclerosis [36]. The other circRNA circ_0062491 was found to be up-regulated in gingival tissues by circRNA sequencing followed with qRT-PCR confirmation and was identified as the sponge of miR-584 [37]. Presently, there are a few reports about the inflammation progression in DPSCs. More so, another comparative research focused on mRNA profile to examine the DPSCs differentiation after TNF-α (10 ng/ml) treatment for 7 (or 14) days [38]. The present study is the first to report that 1195 circRNAs expression had been altered in TNF-α (20 ng/ml) treated DPSCs than normal control DPSCs. These results provide a new gene for additional investigation.

Pulpitis often occurs alongside inflammation, so we focused on differential expressed circRNAs related signaling pathways, including TNF, NF-κB, NLRP3 inflammasome, MAPK, and Wnt signaling pathways [25–28]. Generally, 11 circRNAs were discovered, and just seven circRNAs were confirmed to possess circular structure. Among these, five circRNAs expressions were further confirmed in RNA sequencing samples, and the outcome was comparable with RNA sequence results, in which the expression of hsa_circ_0001978 and hsa_circ_0004417 varied between pulpitis samples and control samples derived from patients with orthodontic.Hsa_circ_0001978 derived from TCONS_l2_00001804 (a lincRNA) [39]. Additionally, earlier studies have verified the role of circ_0001944 obtained from TCONS_l2_00030860 (a lincRNA) in non-small cell lung cancer proliferation via sponging with miR-142-5p [40]. So, hsa_circ_0001978 also plausibly perform a crucial function in pulpitis by behaving like a sponge. For hsa_circ_0004417, its expression was down-regulated in lung adenocarcinoma and patients with atrial fibrillation but up-regulated during the differentiation of human umbilical cord-derived mesenchymal stem cells into cardiomyocyte-like cells [41–43]. Nevertheless, there is no report that investigated the role of hsa_circ_0001978 and hsa_circ_0004417.

Then based on circRNAs (hsa_circ_0001978 and hsa_circ_0004417)-miRNA-mRNA linkage, we discovered that these two circRNAs might regulate the inflammation progression of pulpitis via MAPK and Wnt signaling pathways. Earlier studies have shown that DPSCs perform two roles in pulpitis [3]. On the one hand, DPSCs can identify the invading microbes and then facilitate innate immune feedback to exude distinct inflammation-related factors, such as TNF-α, and finally recruit immune cells to kill invading microbes. On the other hand, DPSCs can move to injured sites to act as a repairer. In these biochemical reactions, TNF, NF-κB, MAPK, and Wnt signaling pathways were activated [44, 45]. Hence, hsa_circ_0001978 and hsa_circ_0004417 may be involved in the inflammation of pulpitis.

Nevertheless, our study was just an introductory investigation. And as such, additional studies are needed to verify whether hsa_circ_0001978 and hsa_circ_0004417 can influence MAPK and Wnt signaling pathways in pulpitis in vivo and in vitro.

## Conclusion

This study was the first to discover that circRNAs profile was changed in the inflammation progression of TNF-α treated DPSCs. Also, hsa_circ_0001978 and hsa_circ_0004417 may be involved in the inflammation of pulpitis via MAPK and Wnt signaling pathways. These results have presented more information for additional investigations.

## Supplementary Information

**Additional file 1:** Agarose ﻿gel electrophoresis of 7 circRNAs.

## Data Availability

All data generated or analyzed during this study are included in this published article [and its Additional file 1].
