# Supplementary figures and images for: Analysis of circRNAs profile in TNF-α treated DPSC
Source: BMC Oral Health. 2022 Jul 3;22:269. doi: 10.1186/s12903-022-02267-2 (PMC9251952; doi:10.1186/s12903-022-02267-2)

has\_circ\_0001658

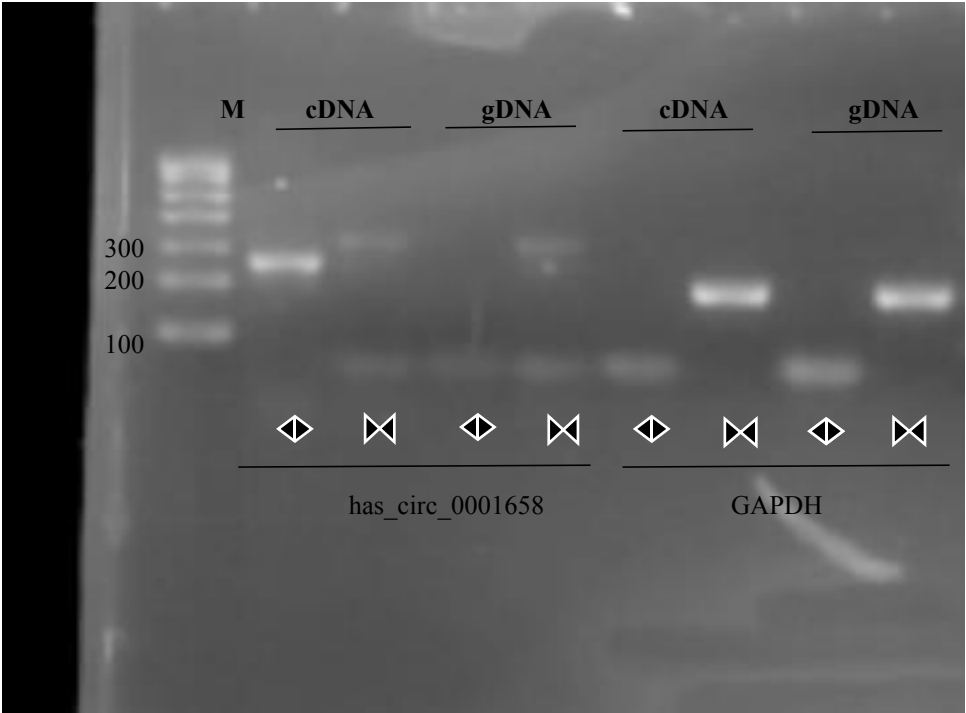

hsa\_circ\_0001978

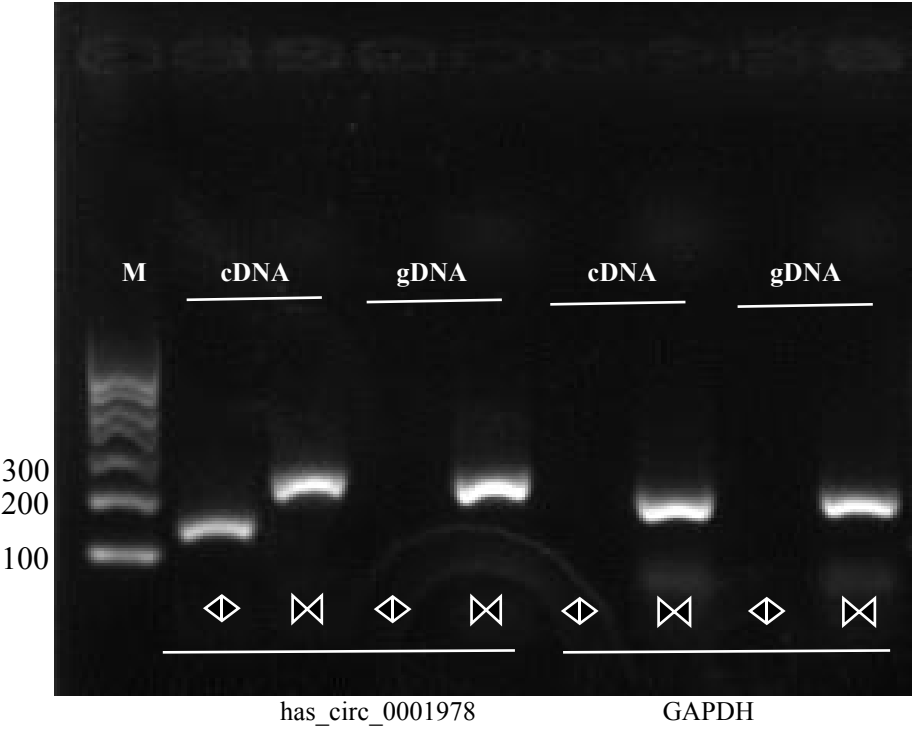

hsa\_circ\_0003910

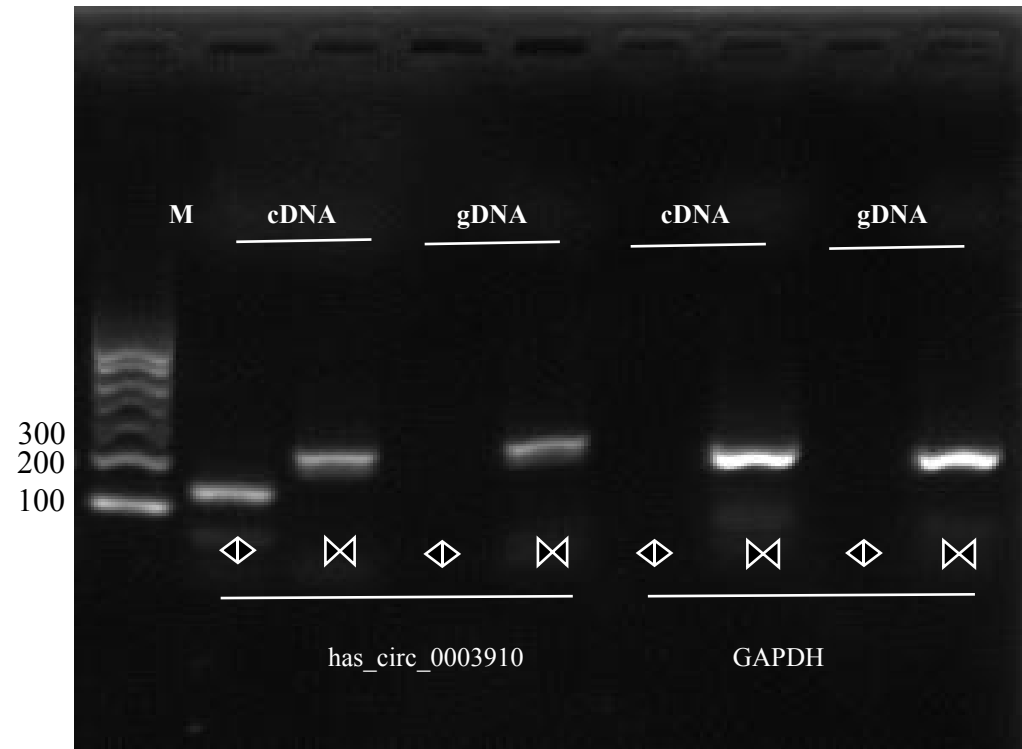

hsa\_circ\_0004314

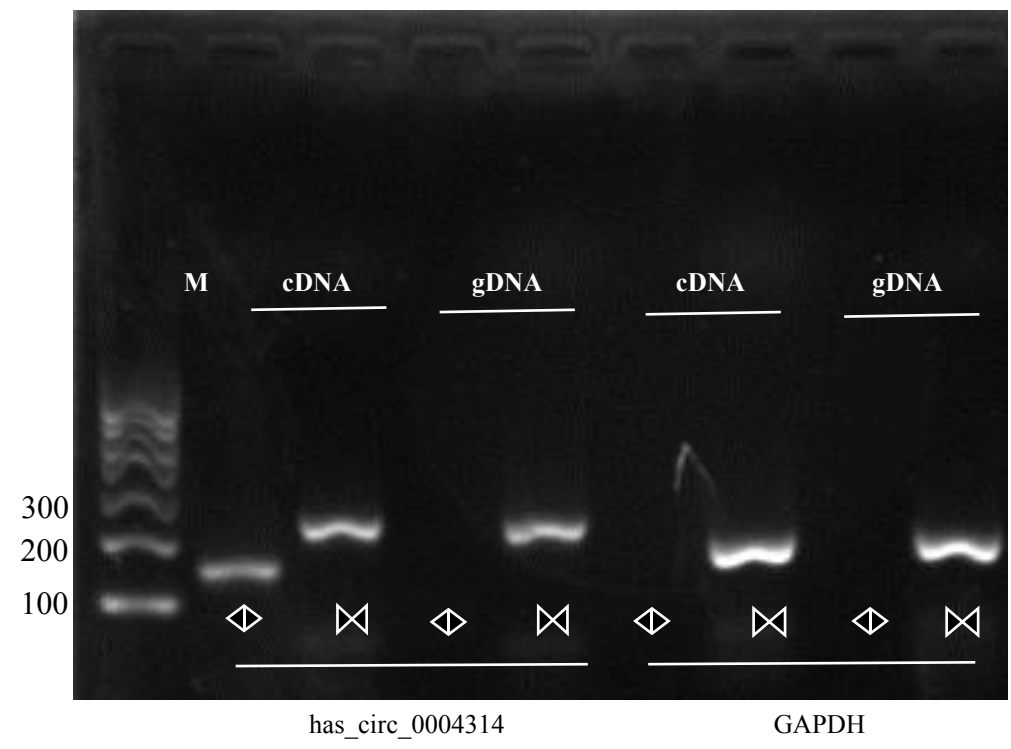

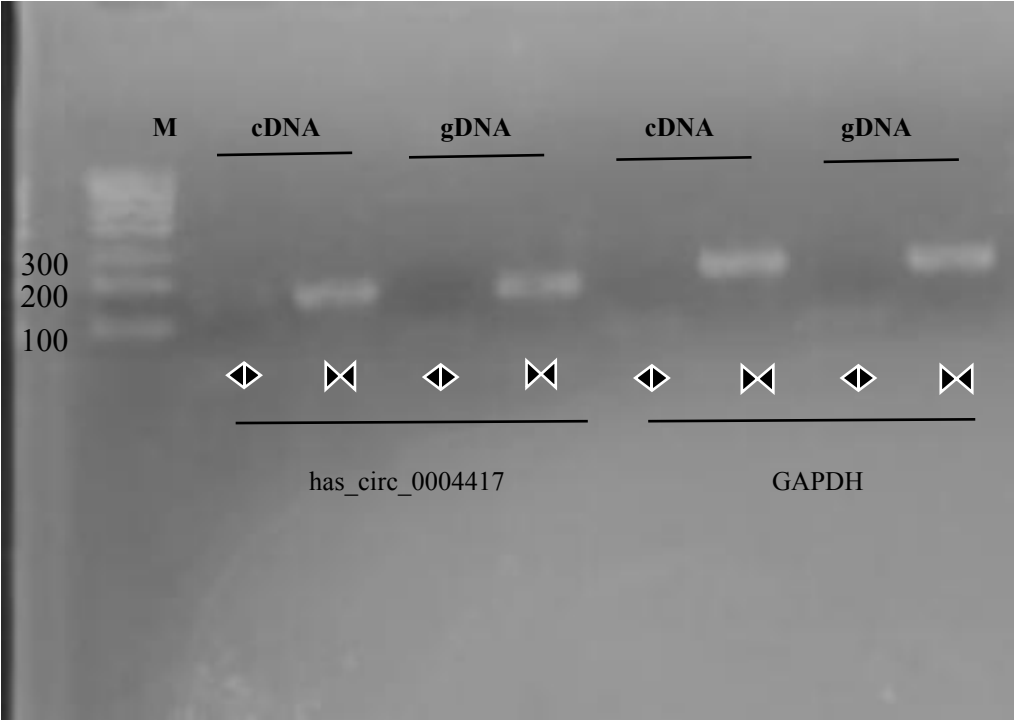

hsa\_circ\_0035915

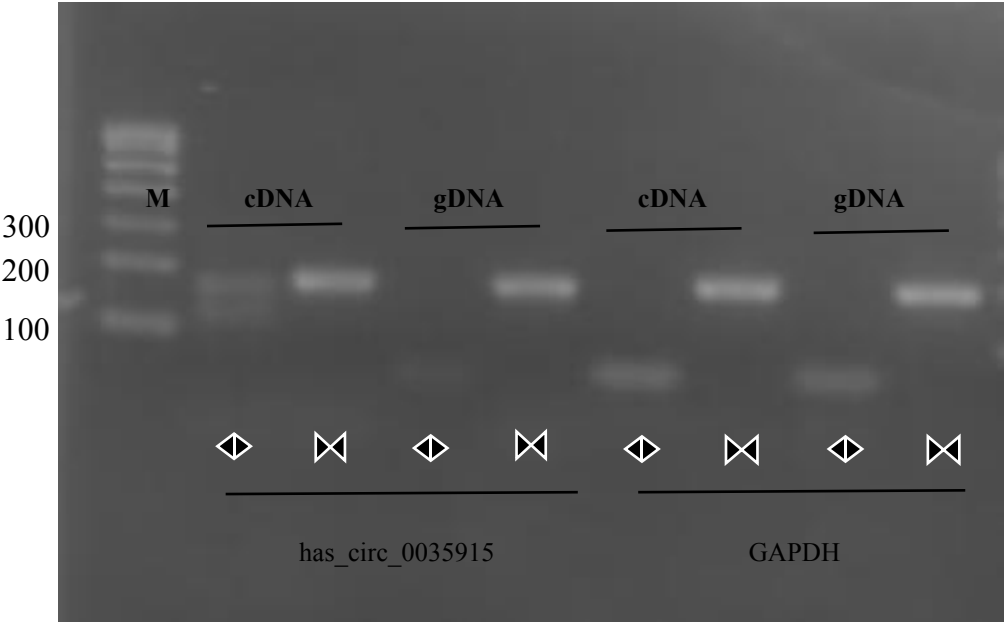

hsa\_circ\_0002545

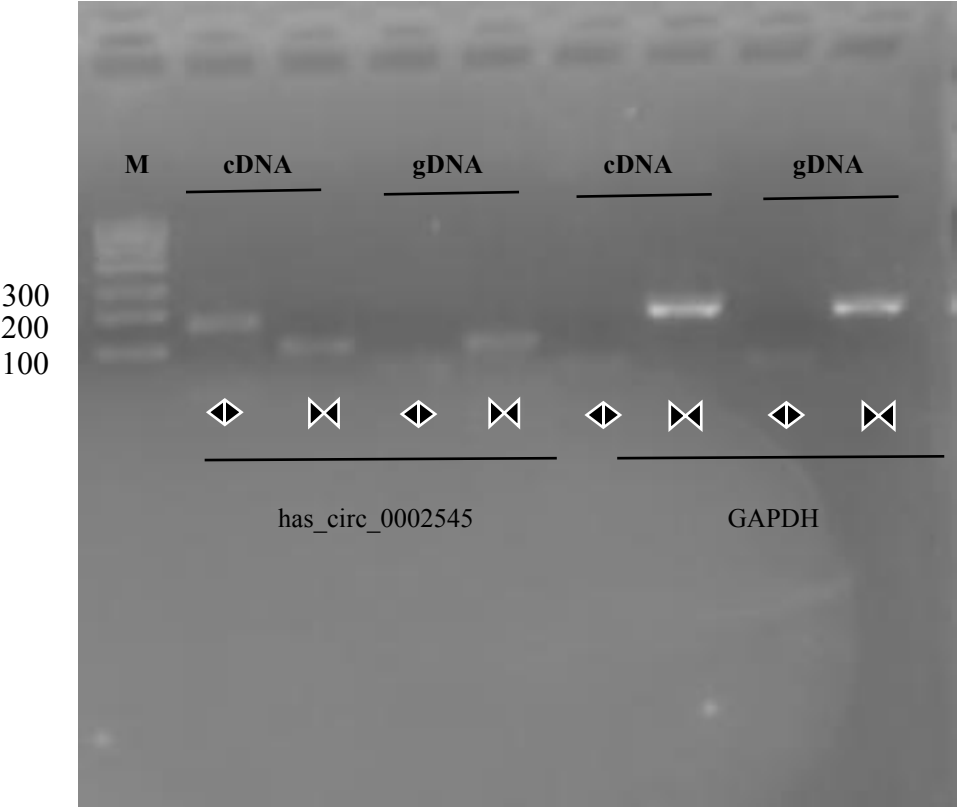

Supplement: Supplementary file 1 — Additional file 1: Agarose ﻿gel electrophoresis of 7 circRNAs. [file 12903_2022_2267_MOESM1_ESM.pdf]
